# Solution characterization of TrhW, a type 4 secretion system regulatory protein from the R27 plasmid

**DOI:** 10.1063/4.0001225

**Published:** 2026-10-19

**Authors:** Christina S. Rodriguez, Gerald F. Audette

**Affiliations:** Department of Chemistry & Centre for Research on Biomolecular Interactions, York University, 4700 Keele St., Toronto, Ontario M3J 1P3, Canada

## Abstract

Type IV secretion systems (T4SSs) are multiprotein nanomachines that mediate conjugative DNA transfer and contribute to the dissemination of antimicrobial resistance genes. The IncHI1 plasmid R27 encodes TrhW, a periplasmic T4SS-associated protein homologous to the F-plasmid proteins TraW and TrbC, which are expressed as separate polypeptides in many F-like systems but are fused into a single protein in IncHI1 plasmids. Despite its proposed role in conjugation, the structural organization of TrhW has remained unknown. Here, we employed complementary computational and solution-based biophysical approaches to characterize the oligomeric state, architecture, and conformational properties of TrhW. Size-exclusion chromatography (SEC) coupled with multi-angle light scattering demonstrated that TrhW exists as a monomer in solution, while circular dichroism spectroscopy revealed a structured protein containing substantial non-canonical secondary structure consistent with flexible regions. SEC-SAXS (small-angle x-ray scattering) showed that TrhW adopts an elongated, non-globular architecture with a radius of gyration of 44.6 Å and a maximum particle dimension of 160 Å. *Ab initio* reconstructions, BilboMD, and ensemble optimization modeling demonstrated that the experimental scattering data are best described by an ensemble of interconverting conformations rather than a single static structure. Although AlphaFold accurately predicts the overall domain organization of TrhW, the solution-state analyses reveal substantial conformational heterogeneity arising from both the N-terminal region and inter-domain motion. Together, these findings establish TrhW as a dynamic multidomain protein and provide the first experimental structural characterization of this conserved T4SS component, offering a framework for future studies investigating its role in IncHI1 conjugation.

## I. INTRODUCTION

Horizontal gene transfer (HGT) is a process of genetic exchange between donor and recipient cells, facilitating rapid pathogen adaptation and driving evolution and antibiotic resistance.^1^ Conjugation is one of the primary mechanisms of HGT. Plasmids from the *Enterobacteriaceae* family are classified into incompatibility (Inc) groups based on their ability to coexist within the same cell; plasmids belonging to the same Inc group cannot be stably maintained.^2,3^ Inc plasmids are characterized by the type of pili they produce, and a thick and flexible H-pilus defines the IncH group.^4^ IncH conjugative plasmids (IncHI and IncHII) are associated with a variety of multidrug resistance (MDR) phenotypes observed in a range of pathogenic bacteria. The IncHI subgroup is further divided into IncHI1, IncHI2, and IncHI3 based on DNA homology.^5^ In this study, we focus on the prototypical IncHI1 plasmid R27 from *Salmonella enterica* serovar Typhi.

*S. typhi* is the causative agent of typhoid fever, a life-threatening infection transmitted through contaminated food and water. This MDR pathogen remains a public health risk in developing countries with inadequate water infrastructure and poor sanitation. The MDR genes expressed by these pathogens is almost exclusively transferred by the IncHI1 plasmid, such as R27.^6,7^ Conjugative transfer mediated by R27 is thermoregulated, with optimal transfer efficiency between 22 and 30 °C and repression at or above 37 °C, conferring a selective advantage in aquatic and soil environments.^8,9^ The R27 conjugative genes can be divided into two transfer regions, *tra1* and *tra2.*^10^ The *tra*1 region encodes proteins required for mating pair formation (Mpf), DNA transfer replication (Dtr), and a coupling protein.^11^ The *tra*2 region encodes additional proteins for Mpf, the majority of which are essential for H-pilus biogenesis. Although the general function of these proteins is known, the specific roles of the individual components within the system remain poorly defined.

Among the proteins encoded within the R27 conjugative system, TrhW is predicted to play a role in its type IV secretion system (T4SS); however, its structural and biophysical properties (oligomeric state, conformational organization, and potential flexibility of TrhW in solution) remain poorly understood. Here, we employ a combination of size-exclusion chromatography coupled with multi-angle light scattering (SEC-MALS), circular dichroism (CD) spectroscopy, thermal denaturation, and small-angle x-ray scattering (SAXS) to define the solution behavior of TrhW. Our results reveal that TrhW exists as a monomer with a structured core and an elongated, partially flexible architecture, providing insights into its potential functional role within the T4SS.

## II. MATERIALS AND METHODS

### A. Cloning, protein expression, and purification

The *trhW* gene (aa 28–502) was synthesized by BioBasic into the pET28a(+) vector and was transformed into chemically competent *Escherichia coli* cells by heat shock. Cloning was verified by Sanger sequencing at the Center for Applied Genomics (The Hospital for Sick Children, Toronto, ON). TrhW was expressed in *E. coli* BL21(DE3) cells grown with shaking (200 rpm, 37 °C) in Luria–Bertani (LB) medium containing 50 *μ*g/ml kanamycin until mid-log phase; protein expression was induced with 1 mM IPTG (isopropyl-β-thiogalactopyranoside) and allowed to proceed overnight at 18 °C. Cells were harvested by centrifugation (5000 × g, 40 min, 4 °C) and resuspended in loading buffer (20 mM Tris pH 7, 200 mM NaCl, 10% glycerol) containing 1 mM PMSF (phenylmethylsulphonyl fluoride). Lysis was performed by sonication on ice at 25% amplitude with cycles of 15 s on and 30 s off, for a total on time of 10 min, following which the soluble fraction was separated from cellular debris by centrifugation (25 000 × g, 40 min, 4 °C).

Tagged protein was purified on a Ni^2+^-NTA agarose column (Cytiva) pre-equilibrated with loading buffer and eluted with buffer plus 500 mM imidazole; sample purity was assessed by SDS-PAGE (Sodium Dodecyl Sulfate–Polyacrylamide Gel Electrophoresis). Further purification by size-exclusion chromatography (SEC) was performed on a HiPrep 16/60 Sephacryl S-100HR column (Cytiva) to remove impurities and to buffer exchange into 10 mM HEPES 4-(2-hydroxyethyl)-1-piperazineethanesulfonic acid), pH 7.0, and 100 mM NaCl. Protein quantification was performed using a bicinchoninic acid (BCA) assay or the Edelhoch method.^12^ Samples were concentrated with a 30 kDa molecular weight cutoff (MWCO) centrifugal concentrator.

### B. Sequence alignments

Sequence alignment of TraW and TrbC proteins was done using Clustal Omega and visualized in Jalview 2.^13,14^ TraW and TrbC can be expressed independently in some F-like systems such as F (*Escherichia coli*), R100 (*Shigella flexneri*), and pED208 (*Salmonella typhimurium*); other systems like R27 (*Salmonella typhimurium*) and SXT (*Vibrio cholerae*) express both in a single polypeptide. The alignment was processed using the following Uniprot accession numbers: F_TraW (P18472); F_TrbC (P18473); R100_TraW (Q7AK66); R100_TrbC (Q9WTC4); pED208_TraW (Q8KNK9); pED208_TrbC (Q8KNK6); R27_TrhW (Q9RGV6); SXT_TraW (A0A0D5XRG0). Pairwise sequence identity values were calculated in Jalview over the aligned TraW-like and TrbC-like regions of R27 TrhW by restricting the analysis to the corresponding conserved blocks identified in the multiple sequence alignment, and values are reported for each homolog relative to the selected region in Table I.

**TABLE I.** Pairwise sequence identity between R27 TrhW and representative TraW and TrbC homologs was calculated over the aligned TraW-like and TrbC-like regions indicated in Fig. 1.

Comparison	Region in R27_TrhW	% Identity
**TrbC homologs**		
R27_TrhW vs F_TrbC	103–312	20%
R27_TrhW vs pED208_TrbC	96–134	20%
R27_TrhW vs R100_TrbC	98–312	21%
**TraW homologs**		
R27_TrhW vs F_TraW	280–496	25%
R27_TrhW vs pED208_TraW	280–479	27%
R27_TrhW vs R100_TraW	280–496	25%

### C. Circular dichroism

The molar ellipticity was measured by acquiring a CD spectrum between 200 and 260 nm using a Jasco J-815 CD spectrophotometer with a path length of 0.1 cm. Collection was done at 22 °C with a 10 *μ*M protein sample in 10 mM KH_2_PO_4_ pH 7.5, 100 mM NaF, 5% glycerol. Following subtraction of the solvent spectra, CD data of TrhW were deconvoluted using BeStSel to determine the secondary-structure content.^15^ A thermal denaturation profile was collected at a single wavelength (222 nm for α-helices) with temperature increasing from 35 to 90 °C at a rate of 1 °C/min. All experiments were collected in duplicate, and the mean of these replicates is recorded.

### D. SEC-MALS–SAXS

SEC-MALS and SAXS data were collected at the BioCAT facility, Sector 18, Advanced Photon Source (Argonne National Laboratory, USA). TrhW (purified as per Sec. II A) samples (230 *μ*l at 5 mg/ml) were injected onto a Superdex 200 10/300 Increase SEC column (Cytiva) using an Agilent Infinity II HPLC at a flow rate of 0.6 ml/min. Sequential in-line MALS analysis was performed with a DAWN Heleos II instrument and an Optilab T-rEX direct refractive index (dRI) detector (Wyatt). SAXS data (Table II) were collected using an Eiger2 XE 9M detector at a sample-to-detector distance of 3.7 m and a wavelength of 0.1033 nm.^14,15^ The data were buffer subtracted using frames 662–742, and the elution peak was isolated using frames 1320–1364 prior to further analysis.

**TABLE II.** SEC-MALS–SAXS data collection parameter for TrhW.

Data collection parameters	TrhW
Instrument	BioCAT (Sector 18, APS)
Detector	Eiger2 XE 9M
Wavelength (Å)	1.033
q-measurement range (1/Å)	0.0029–0.42
Exposure time (s)	0.5
Size exclusion column	Superdex 200 10/300 Increase
Flow rate (ml/min)	0.6
Temperature (°C)	20
Coflow buffer	10 mM K_2_HPO_4_, 100 mM KCl, pH7.0
**Sample conditions**	
Concentration (mg/ml)	5.0
Loaded volume (*μ*l)	230
Buffer	20 mM HEPES, 100 mM NaCl, pH 7.0
**Data analysis**	
Buffer averaging range (frames)	662–742
Sample averaging range (frames)	1320–1364 (45 frames)
**Structural parameters**	
*I*(0) (cm^−1^) [from P(r)]	0.0163 ± 1.20 × 10^−5^
R_g_ (Å) [from P(r)]	45.0 ± 0.07
*I*(0) (cm^−1^) (from Guinier)	0.0164 ± 1.54 × 10^−5^
R_g_ (Å) (from Guinier)	44.6 ± 0.13
Guinier fit range, q (Å^−1^)	0.0031–0.0219
qR_g_ range	0.14–0.98
D_max_ (Å)	160
**Molecular-weight analysis**	
Bayes (kDa)	59.5
V_c_ (kDa)	59.4
V_p_ (kDa)	56.7

SAXS data collection and analysis parameters for R27 TrhW are summarized in Table II, which provides details of the experimental conditions and data processing, including buffer composition, exposure parameters, and primary scattering-derived values such as the radius of gyration (R_g_) and maximum particle dimension (D_max_).^16^

### E. SAXS-based computational modeling

SAXS data were analyzed using RAW and ATSAS software packages.^17,18^ Bead models were generated from 15 independent reconstructions using DAMMIF, averaged and refined with DAMAVER.^19^ Structural clustering of the independent reconstructions was performed using DAMCLUST to assess the reproducibility of the *ab initio* models.^19,20^ Electron density maps were reconstructed with DENSS using 20 aligned and averaged reconstructions.^21^ SAXS data for TrhW were submitted to SASBDB (https://www.sasbdb.org) under accession codes SASDZ4A. Table III summarizes the electron density reconstruction performed using DENSS, including reconstruction parameters and quality metrics.

**TABLE III.** SEC-MALS–SAXS electron density reconstruction for TrhW using DENSS.

	TrhW
Mode	Slow
Symmetry	False
Number of reconstructions	20
Averaged	True
Refined	True
Mean real space correlation (RSC)	0.753 ± 0.055
Included models	19 of 20
R_g_	42.7
Chi^2^	2.84
Resolution (Å)	47.01

Ensemble analysis was performed using BilboMD to evaluate conformational heterogeneity in solution. Multi-state models were generated from the AlphaFold (AF) starting model and assessed based on agreement with experimental SAXS data using χ^2^ minimization.^22,23^ Theoretical scattering profiles for the AF-predicted TrhW model were calculated using CRYSOL (ATSAS) and fitted to the experimental SAXS data to assess agreement and generate residual plots.^24^ All models were rendered using ChimeraX.^25^

To further investigate conformational heterogeneity in solution, ensemble optimization method (EOM) analysis was performed using the ATSAS package online (v3.0).^26,27^ The experimental SAXS profile obtained for TrhW was used as input for ensemble modeling. The initial pool of conformers were generated using RANCH based on the TrhW AF structural predictions. Several models were evaluated including unconstrained and differing constrained ensembles. The final model used residues 164–286 and 324–475 as independent rigid bodies, while residues 1–163 and 287–323 were defined as flexible segments. Pools consisting of 10 000 conformers were generated and optimized using the genetic algorithm GAJOE to identify ensembles that best reproduced the experimental SAXS profile. The quality of the resulting fits was assessed using χ^2^ values, and the distributions of R_g_ and D_max_ were analyzed to evaluate conformational variability.

## III. RESULTS AND DISCUSSION

### A. TrhW represents a conserved TrbC–TraW fusion protein

Despite differences in plasmid classification, T4SS components are often functionally conserved across incompatibility groups. IncHI1 plasmids encode TrhW, whereas IncF plasmids encode the related protein TraW, along with its interaction partner TrbC, supporting a shared role for these proteins in coordinating T4SS assembly and/or substrate transfer. Sequence alignments of these proteins across diverse conjugative plasmids (Fig. 1) highlight a conserved interaction interface between TraW and TrbC, which is recapitulated in TrhW as a single polypeptide.^28^ The fusion architecture observed in TrhW is particularly notable because TraW and TrbC are encoded as separate proteins in many F-like systems, where they are proposed to function together during the T4SS assembly. Fusion of these homologous components into a single polypeptide may preserve functional interactions while eliminating the requirement for intermolecular association, potentially altering both structural organization and conformational dynamics. It is important to note that the sequences used have the signal sequence portion removed; the TrhW construct used throughout this study corresponds to residues 28–502 and therefore represents the predicted mature periplasmic form of the protein following signal peptide cleavage. To further investigate the structural and functional implications of this conserved architecture, we performed biophysical and structural characterization of TrhW.

**FIG. 1.**
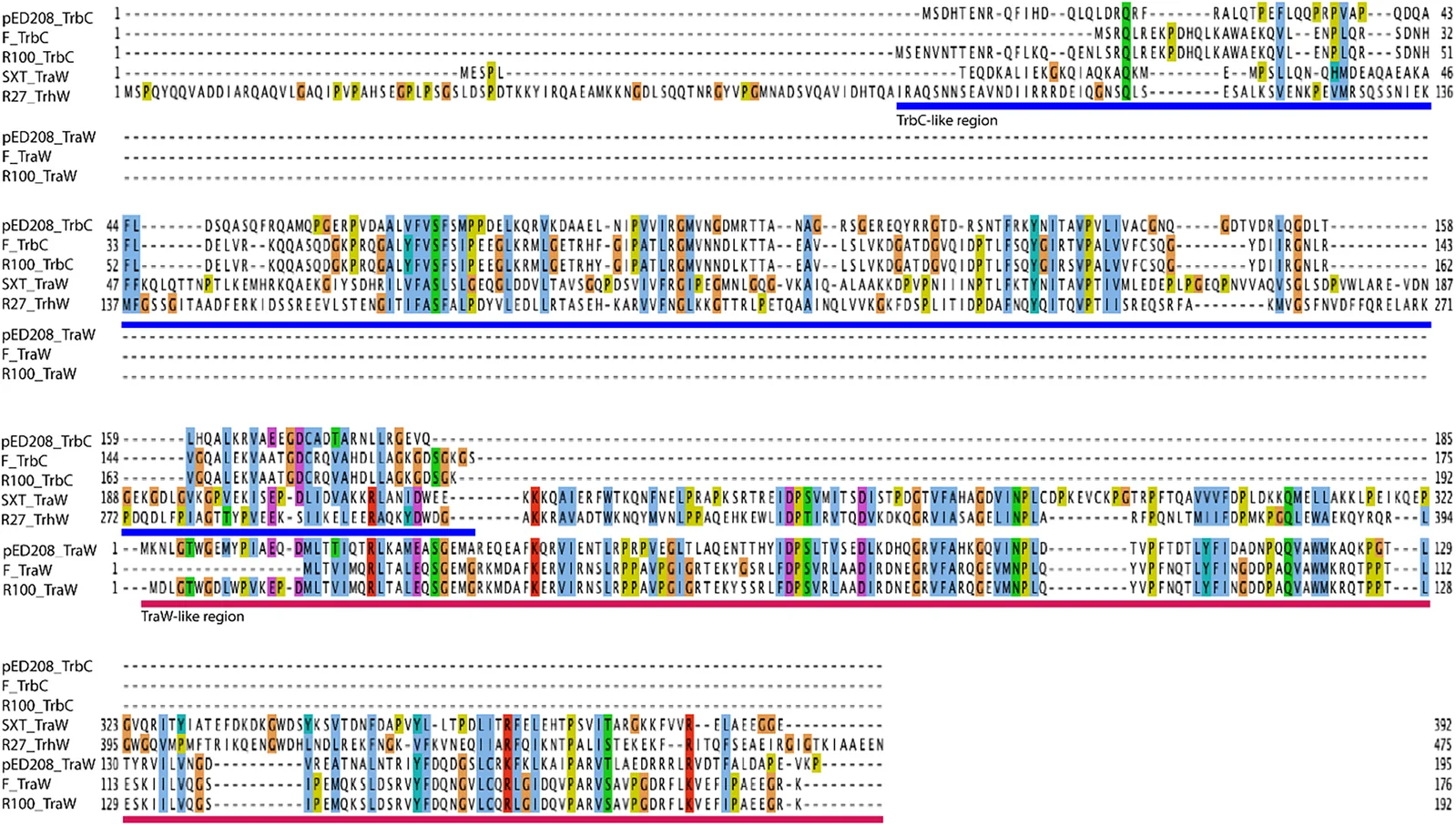
Multiple sequence alignment of R27 TrhW with representative TraW and TrbC homologs. The alignment was generated using Clustal Omega and visualized in Jalview, with residues colored according to the Clustal scheme. Regions of R27 TrhW aligning with TrbC and TraW homologs are indicated, supporting a fused domain architecture.

A structural prediction of TrhW using AlphaFold (AF) revealed regions of varying confidence, with notably lower confidence observed in the N-terminal region, suggesting potential conformational flexibility [Fig. 2(a)].^29^ AlphaFold's predicted local distance difference test (plDDT), which provides a per-residue metric based on expected local interatomic distance accuracy, was used to assess model reliability.^30^ plDDT scores range from very low to low confidence for the first ∼160 residues; this is supported by the predicted aligned error (PAE), which also indicates elevated error in this region [Fig. 2(b)]. The PAE estimates the confidence in the relative positioning of residues and domains within the model.^31^ In contrast, the remaining residues predominantly exhibit high to very high plDDT scores, suggesting reliable local structural prediction. However, the corresponding PAE displays a mixture of intermediate to high error, indicating uncertainty in the relative orientation of these regions. Together, these observations suggest that while local domain structures are likely predicted accurately, their relative arrangement may be less defined, consistent with the presence of inter-domain mobility and conformational heterogeneity within TrhW. Importantly, these predictions alone cannot distinguish between intrinsic disorder, domain motion, or the presence of multiple structured conformational states. Consequently, experimental characterization is required to determine whether the low confidence regions predicted by AlphaFold correspond to genuine conformational flexibility in solution.

**FIG. 2.**
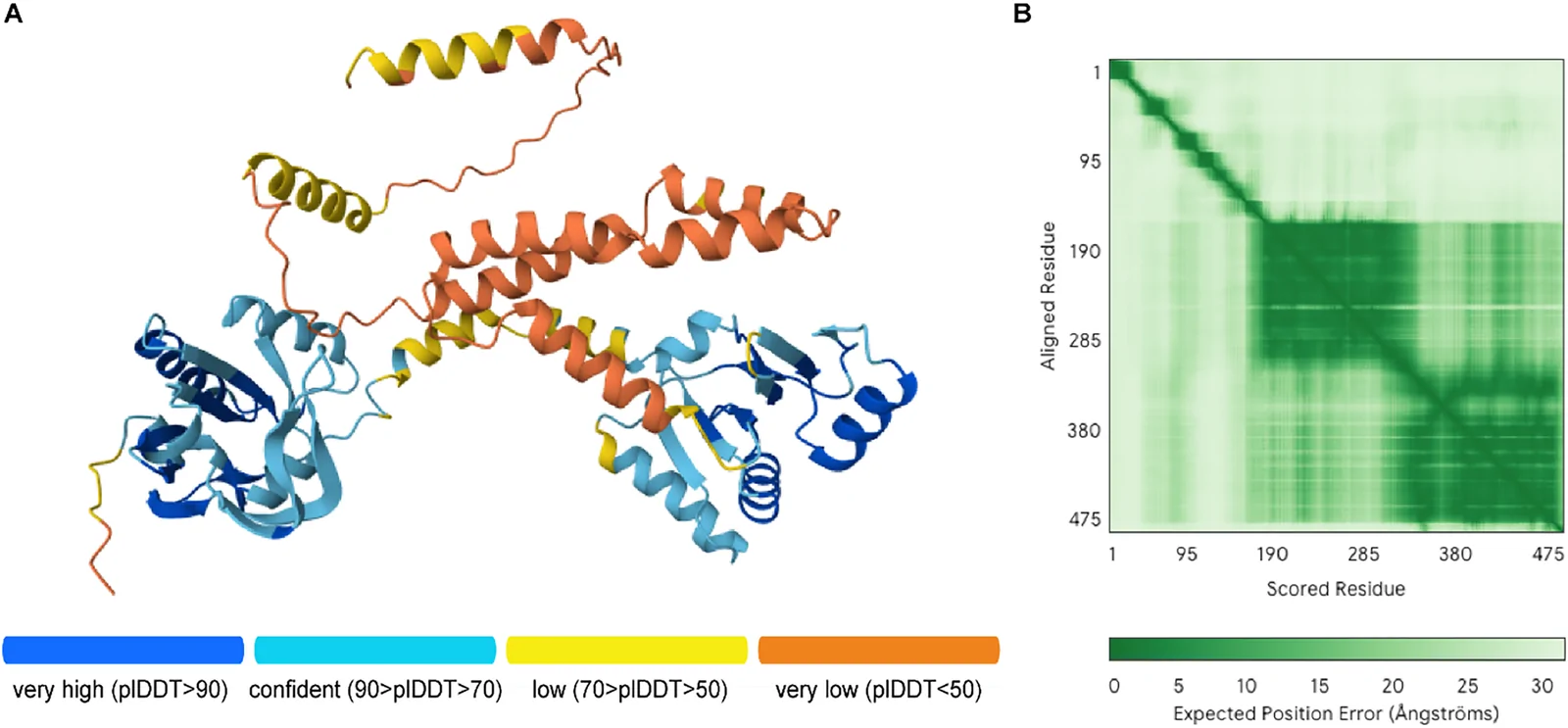
(a) AlphaFold (AF) predicted structure of TrhW, with color legend to indicate regions of very high confidence to very low confidence based on the plDDT score. (b) Predicted aligned error of the TrhW model. Higher expected error is depicted in light green, and as the error decreases, the color darkens. The PAE indicates a high expected error in the N-terminal region.

### B. TrhW is monomeric in solution and contains both ordered and flexible structural elements

To assess the oligomeric state of TrhW in solution, the SEC-MALS analysis was performed, revealing a single monodisperse species consistent with a monomeric assembly [Fig. 3(a)].^32^ TrhW eluted as a single SEC peak with a slight asymmetry on the trailing edge. The MALS-derived molecular mass showed some variation across the elution peak; however, analysis of the defined peak region (21.73–23.48 min) yielded an average molecular mass of 57.5 kDa, in close agreement with the expected monomeric mass of 57.3 kDa. The calculated M_w_/M_n_ of 1.001 further indicated low molar-mass dispersity within the analyzed peak. Together, these results support TrhW existing predominantly as a monomer in solution. Notably, although TrhW contains regions homologous to both TraW and TrbC, which exist as separate proteins in IncF plasmids, fusion of these domains does not promote self-association in solution.^28,33^ Instead, TrhW behaves as a discrete monomer under the conditions examined, suggesting that the incorporation of both functional regions into a single polypeptide is sufficient to maintain structural integrity without requiring oligomerization. Having established TrhW's oligomeric state, CD spectroscopy was employed to assess its secondary-structure content and stability [Figs. 3(b) and 3(c)].

**FIG. 3.**
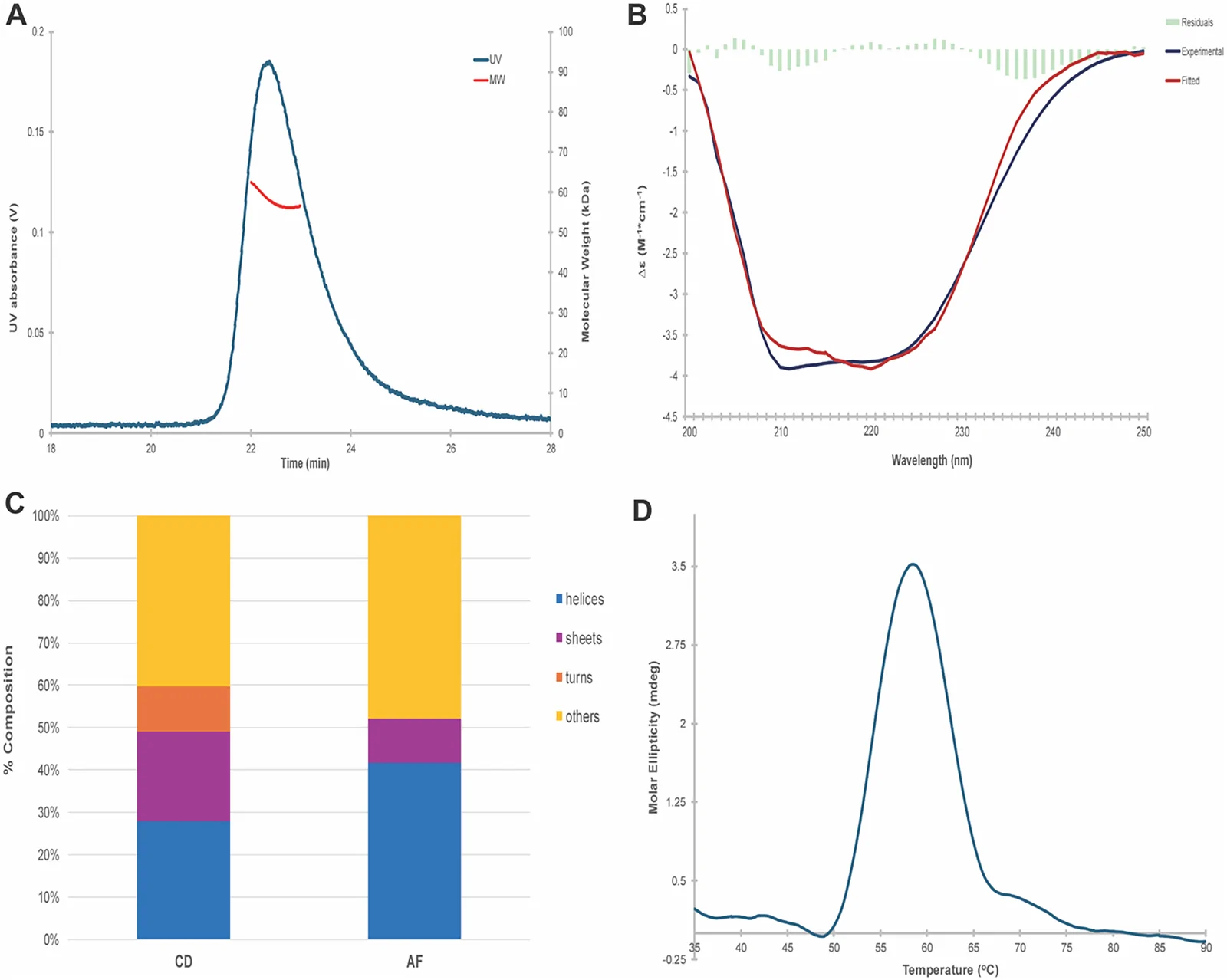
(a) SEC-MALS analysis of TrhW shows a single monodisperse peak with a molecular weight of 57–58 kDa, consistent with the theoretical monomer mass (57.5 kDa). (b) Circular Dichroism (CD) spectra expressed in delta epsilon. The fitted CD spectrum was obtained by least squares deconvolution of the experimental data into a linear combination of reference spectra representing different secondary-structure elements, with residuals reflecting the difference between experimental and calculated ellipticity. (c) Secondary-structure composition of TrhW predicted by both CD and AF. (d) Thermal melt of TrhW monitored by molar ellipticity. TrhW experienced a melting temperature of 58 °C.

The experimental CD data were deconvoluted into a linear combination of reference spectra representing distinct secondary structure elements using BeStSel.^34^ BeStSel decomposes the signal into contributions from α-helices, β-sheets, turns, and other. Unlike traditional deconvolution methods, BeStSel distinguishes between different β-sheet geometries, including parallel and antiparallel arrangements, allowing for a more detailed and accurate representation of β-structure content.^15,35^ This expanded classification avoids forcing structurally diverse regions into simplified α/β categories and often results in a larger “other” fraction. The “other” component encompasses loops, irregular structures, distorted secondary elements, and flexible regions and represents a genuine structural contribution rather than noise.^35^ For the purpose of this study, the β-sheet contributions were combined into a single fraction.

The resulting fitted spectrum closely reproduces the experimental data, with minimal residuals (defined as the difference between experimental and fitted values), indicating a good agreement between the model and the observed signal [Fig. 3(b)]. Following deconvolution in BeStSel, the secondary-structure composition of TrhW was estimated as 28% α-helical, 21.1% β-sheet, 10.6% turn, and 40.3% other. In comparison, AF predicts a composition of 41.6% α-helical, 10.5% β-sheet, and 47.9% others [Fig. 3(c)]. While AF predicts increased α-helical content and reduced β-sheet contribution relative to the experimental data, the substantial “other” fraction observed in both analyses indicates that a significant portion of the protein does not adopt canonical secondary structure. Notably, the 40.3% “other” fraction corresponds to approximately 160 residues of the 475-residue construct, closely matching the ∼160-residue N-terminal region identified by AlphaFold as having low confidence and elevated PAE values [Fig. 2(b)]. Although CD spectroscopy cannot localize structural disorder to specific regions of the sequence, the agreement between these independent analyses supports the presence of substantial non-canonical structure within the N-terminal portion of TrhW. The overall agreement between the two approaches supports a model in which TrhW contains both ordered structural elements and regions exhibiting increased conformational freedom. Importantly, however, the CD measurements provide experimental evidence that extends beyond the AlphaFold prediction by demonstrating that these features persist in solution rather than arising solely from computational uncertainty. The comparison of CD-derived secondary structure with AF predictions therefore provides useful cross-validation of overall structural content, although such comparisons should be interpreted with caution, as CD reports ensemble-averaged solution behavior, whereas AlphaFold represents a single predicted conformation and may overestimate ordered structure in flexible regions.

### C. Thermal stability of TrhW

To further evaluate the stability of TrhW, thermal denaturation was monitored by CD by tracking changes in molar ellipticity as a function of temperature [Fig. 3(d)]. Thermal unfolding produced a sigmoidal transition with an apparent melting temperature (Tm) of approximately 58 °C, indicating moderate thermal stability. The presence of a well-defined transition suggests that the protein contains a thermally stable folded core despite the presence of regions predicted to exhibit structural flexibility. Although cooperative unfolding is often associated with compact globular proteins, the CD signal monitored at 222 nm primarily reflects changes in ordered secondary structure.^36^ Consequently, the observed transition is likely dominated by unfolding of the structured domains within TrhW, whereas the flexible N-terminal region, which is predicted to contain substantial non-canonical structure and contributes comparatively less to the CD signal, may remain conformationally heterogeneous throughout the experiment. This interpretation is consistent with the SAXS and ensemble modeling results, which indicate that TrhW contains a stable structural framework connected to flexible regions rather than behaving as a globally disordered protein. Taken together, the SEC-MALS and CD analyses support a model in which TrhW exists as a monomeric, moderately stable protein containing both folded structural elements and conformationally dynamic regions. To further define the overall size, shape and conformational properties of TrhW in solution, size-exclusion chromatography coupled with small-angle x-ray scattering (SEC-SAXS) analysis was performed.

### D. SEC-SAXS reveals an elongated and conformationally heterogeneous solution structure

SEC-SAXS measurements were collected to provide low-resolution structural information on the size, shape, and conformational properties of TrhW in solution (Fig. 4). The elution profile indicates the presence of a single species, evidenced by a single narrow peak [Fig. 4(a)]. The buffer-subtracted scattering profile [Fig. 4(d)] displays a smooth decay in intensity with increasing q, with no evidence of an upturn at low q, indicating a monodisperse sample free of aggregation. Acceptable signal-to-noise is maintained across the measured q range, supporting the quality of the data and its suitability for subsequent analysis. This is further supported by the linearity of the Guinier plot and the normalized residual plot [Figs. 4(b) and 4(c)], from which a radius of gyration (R_g_) of 44.6 ± 0.13 Å was obtained.^37^ Molecular weight estimation yielded an average mass of ∼58.5 kDa, consistent with the expected monomeric molecular weight of 57.5 kDa. Agreement between the SAXS-derived molecular weight and the SEC-MALS measurements confirms that the scattering data correspond to a monomeric species and exclude higher-order oligomerization as the source of the observed structural dimensions. The R_g_ decreased across the SEC elution peak, indicating variation in the average dimensions of TrhW across the elution profile. A similar trend was observed in the SEC-MALS analysis, where the apparent molecular mass decreased toward the trailing edge of the peak. Given that the overall molecular mass determined by SEC-MALS and SAXS is consistent with predominantly monomeric TrhW, the variation in R_g_ may reflect differences in the hydrodynamic properties of the protein across the elution peak rather than discrete oligomeric states. This behavior is consistent with conformational heterogeneity within the monomeric population, although the SEC-based measurements alone cannot distinguish individual conformational states. The observed R_g_ is larger than would be expected for a compact globular protein of similar molecular weight, suggesting that TrhW adopts an extended or non-globular conformation in solution. Given the absence of aggregation in the scattering profile and the monomeric state confirmed by both SEC-MALS and SAXS molecular-weight analysis, this increased R_g_ is most consistent with an elongated architecture and/or substantial conformational flexibility.

**FIG. 4.**
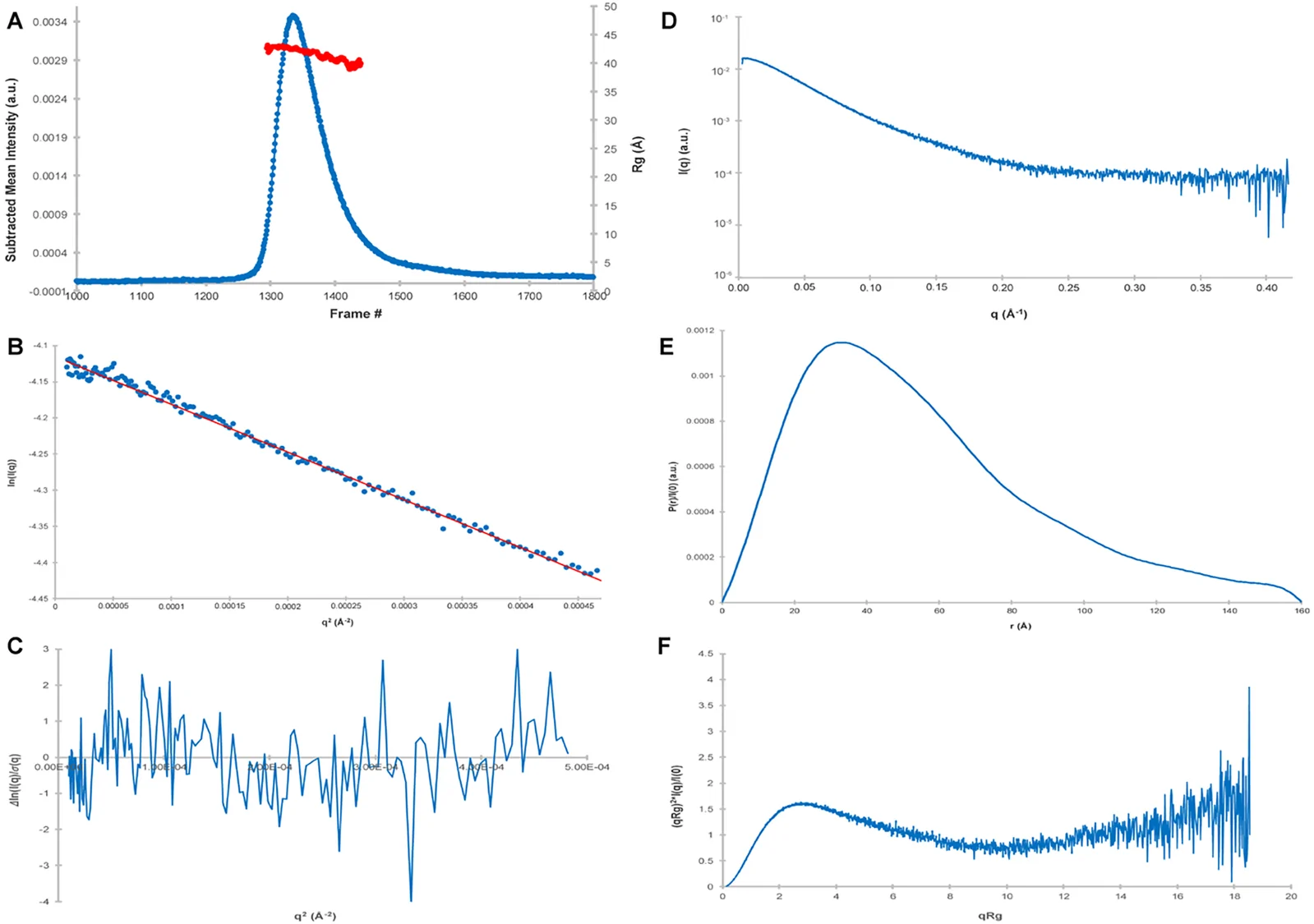
SAXS analysis of TrhW. (a) Series plot of scattering intensity, demonstrating consistency across frames indication absence of radiation damage or aggregation. Buffer subtraction was done using frames 662–742, and the sample averaging was done using frames 1320–1364. Curve indicated in red correlates with R_g_. (b) Guinier analysis of TrhW over the fitting range q = 0.0031–0.0219 Å^−1^, yielding an R_g_ of 44.6 ± 0.13 Å. (c) Normalized residuals corresponding to the Guinier fit shown in (b). (d) Buffer subtracted and averaged scattering profile I(q) plotted on a logarithmic scale as a function of q, showing smooth decay consistent with a monodispersed sample. (e) Pair-distance distribution function P(r) calculated using GNOM, revealing a maximum particle dimension D_max_ of 160 Å and a slightly skewed distribution indicative of an elongated architecture. (f) Dimensionless Kratky plot, displaying a slightly broadened peak with an upward trend at high q, consistent with a partially flexible/extended conformation in solution.

The dimensionless Kratky plot [Fig. 4(f)] provides insight into the conformational properties of TrhW, exhibiting a broadened peak with a slight upturn at high q, consistent with an elongated and non-globular particle. The P(r) distribution [Fig. 4(e)] displayed a skewed curve with an extended tail at high r, further supporting an elongated molecular shape. The distribution also exhibits peak broadening, suggesting that TrhW samples a range of conformations in solution rather than existing as a single rigid structure.^38^ The maximum particle dimension (D_max_) of TrhW was determined to be 160 Å. Importantly, neither D_max_ nor R_g_ can be inferred reliably from AF predictions alone, as these parameters reflect ensemble-averaged properties measured directly in solution. The combination of a large R_g_, extended D_max_, and non-ideal Kratky profile therefore provides experimental evidence that TrhW adopts an elongated architecture with significant conformational heterogeneity under solution conditions. Together, these analyses establish a structural framework for subsequent *ab initio* shape reconstruction and ensemble modeling.

### E. *Ab initio* reconstruction supports conformational heterogeneity in solution

An *ab initio* molecular envelope was reconstructed using DAMMIF, revealing an elongated asymmetric shape consistent with the overall SAXS analysis (Fig. 5).^20^ Structural clustering of the 15 independent DAMMIF reconstructions identified a dominant cluster comprising seven models, which was used to generate the representative averaged envelope shown in Fig. 5. Docking of the AF-predicted model within the SAXS-derived bead envelope shows good agreement for the folded C-terminal core, while the N-terminal region exhibits variable positioning relative to the experimental density. Although the AF model accounts for the overall dimensions of the folded core, it does not fully explain the distribution of experimental density observed within the SAXS envelope, suggesting that a single static conformation may be insufficient to describe the solution behavior of TrhW. This observation is consistent with the presence of conformational heterogeneity and supports the interpretation that the N-terminal region contributes substantially to the extended solution structure.

**FIG. 5.**
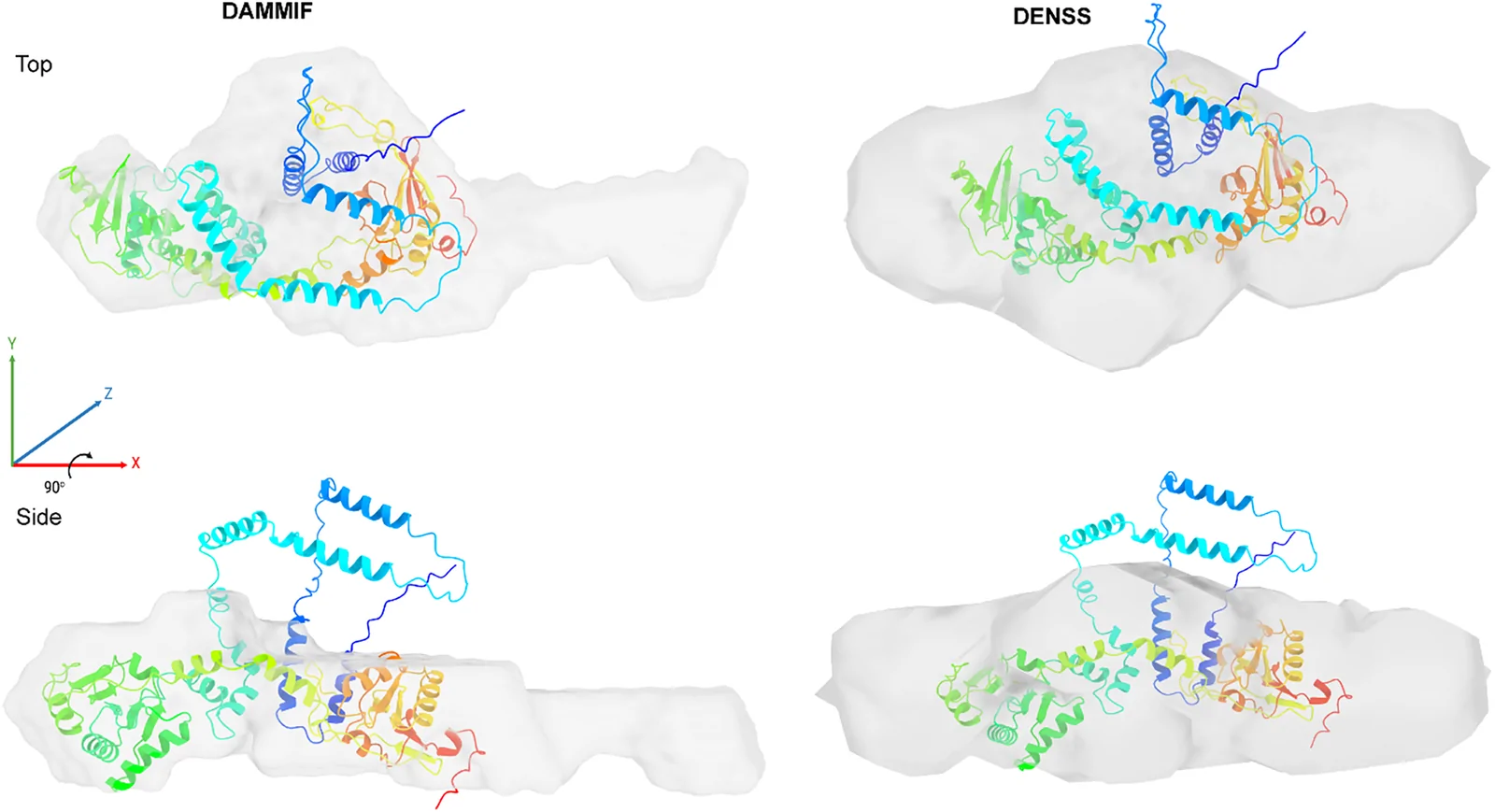
Comparison of SAXS-derived reconstructions of TrhW. Top and side views of the AF-predicted model docked into the *ab initio* reconstruction generated using DAMMIF (left) and the electron density map generated using DENSS (right). The DAMMIF model was displayed using a dummy-atom radius of 4.8 Å, corresponding to the radius specified in the refined dummy-atom model. The DENSS map was displayed at a density cutoff of 1.1 × 10^−4^ and lightly smoothed for visualization (smoothing factor = 0.15, 2 iterations). The DAMMIF model displays an elongated asymmetric envelope with good agreement for the folded C-terminal core. The DENSS reconstruction shows a more diffuse density, with the AF-predicted model only partially occupying the volume, consistent with conformational flexibility, particularly within the N-terminal region.

DENSS was used to reconstruct a three-dimensional electron density map directly from solution scattering data through iterative refinement in real space, enabling a model-independent visualization of the particle's overall shape and internal density distribution.^39^ Docking the AF-predicted model within the electron density reveals that the model does not fully occupy the reconstructed volume, leaving regions of unfilled density (Fig. 5). This discrepancy is particularly informative because it highlights features of the experimental scattering data that are not captured by a single AlphaFold-derived structure. Such behavior is commonly observed for proteins exhibiting conformational heterogeneity, dynamic domain motion, or ensemble averaging in solution rather than a single rigid conformation.^40^

DAMMIF reconstructions often appear more compact or fully occupied because they represent an averaged bead model constrained by predefined parameters and may overestimate occupied volume.^41^ DENSS, in contrast, reconstructs electron density directly from the scattering data without imposing a bead-based representation, thereby highlighting regions where density is consistently supported.^42^ If multiple conformations are sampled in solution, the resulting averaged density can appear more diffuse or partially unoccupied, particularly within flexible regions, giving rise to the observed unfilled volume. Together, the DAMMIN and DENSS reconstructions provide independent experimental evidence that TrhW is best described as a conformationally heterogeneous particle rather than a rigid elongated structure, motivating the subsequent ensemble optimization analysis.

### F. Ensemble modeling reveals multiple solution-state conformers

BilboMD analysis (Fig. 6) revealed that a single structural model was insufficient to describe the experimental SAXS data (χ^2^ = 46.67), with systematic deviations observed across the scattering profile. In contrast, ensemble modeling significantly improved the fit (χ^2^ ≈ 1.37), indicating that TrhW adopts multiple conformations in solution. This improvement demonstrates that the experimental scattering profile is better described by an ensemble of conformations than by a single static structural model. The improved agreement is visually supported by the corresponding fits to the scattering profile, where the ensemble model more closely follows the experimental curve across the full *q* range [Fig. 6(a)].^37^ Comparison of the residual plots further highlights this difference, with the single-structure model showing pronounced systematic deviations [Fig. 6(b)], whereas the ensemble model exhibits smaller residuals that are more randomly distributed about zero [Fig. 6(c)]. BilboMD generated ensemble solutions containing one to five conformers (Table IV). The one-state ensemble yielded a χ^2^ = 34.99, whereas increasing the ensemble size to two conformers improved the fit χ^2^ = 1.94. Ensemble solutions containing three, four, and five conformers also yielded χ^2^ values of 1.50, 1.38, and 1.37, respectively. The five-state ensemble was therefore selected providing the best agreement with the experimental scattering data and is shown in Fig. 6(a). Representative structures from the independently optimized BilboMD solutions with χ^2^ = 1.37 differed primarily in the relative positioning of the N-terminal region with respect to the folded C-terminal core while maintaining the overall domain architecture [Fig. 6(e)]. The two folded domains maintained their overall structures across the ensemble, while their positions and orientations relative to one another varied between conformations. These findings support a model in which TrhW consists of a relatively stable core with localized flexibility, rather than existing as a single rigid structure in solution. Although BilboMD demonstrated that multiple conformations are required to describe the experimental SAXS data, it does not define the specific structural features responsible for this conformational heterogeneity. To further investigate the nature of these solution-state conformations and evaluate which regions of TrhW contribute to the observed flexibility, the ensemble optimization method (EOM) analysis was performed.

**FIG. 6.**
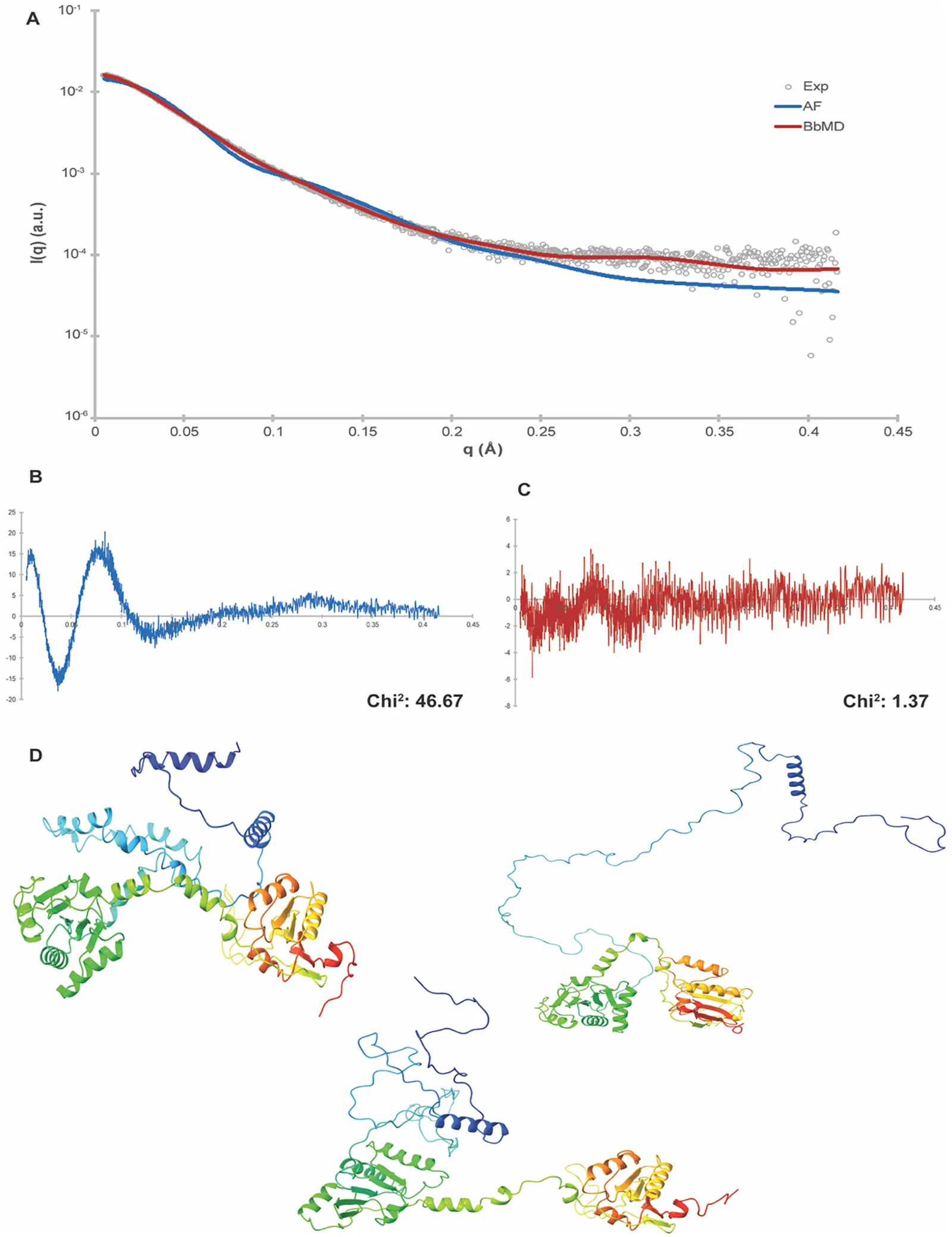
Ensemble modeling of TrhW using BilboMD. (a) Comparison of experimental SAXS data (gray) with scattering calculated from the AF model (blue) and the five-state BilboMD ensemble (red). The five-state BilboMD ensemble provided the best agreement with the experimental data (χ^2^ = 1.37). (b) Residual plot for the AF model, showing systematic deviations from the experimental data (χ^2^ = 46.67). (c) Residual plot for the two-state BilboMD ensemble shown in (a), showing smaller residuals that are more randomly distributed around zero (χ^2^ = 1.37). (d) Representative conformations from the selected five-state BilboMD ensemble, illustrating variation in the relative positions and orientations of the structured domains while maintaining their overall folds.

**TABLE IV.** Summary of BilboMD ensemble modeling of TrhW.

Ensemble size	χ ^2^	Conformer weights
1	34.99	1.000
2	1.94	0.584, 0.416
3	1.50	0.406, 0.189, 0.405
4	1.38	0.452, 0.140, 0.278, 0.130
5	1.37	0.401, 0.272, 0.063, 0.090, 0.175

### G. Ensemble optimization supports inter-domain flexibility

To further investigate the structural basis of the conformational heterogeneity identified by BilboMD, EOM analysis was performed to test whether flexibility within the N-terminal region and relative motion between structured domains could account for the experimental SAXS data. Initial EOM analyses were performed using both unconstrained and constrained ensemble models. In the unconstrained model, all residues were treated as flexible, allowing the protein to sample a broad range of conformations (Table V).^27^ While this model produced a good fit to the experimental SAXS data (χ^2^ = 1.42), the resulting conformers lacked a well-defined structural core and were difficult to reconcile with the AF prediction. To incorporate available structural information from the AF prediction, an additional constrained model was generated in which residues 164–475 were treated as a single rigid body, while the N-terminal region remained flexible. Although this model produced a reasonable fit (χ^2^ = 2.38), further improvement was sought to better account for the flexibility suggested by the PAE analysis. The AF model and the corresponding PAE matrix suggested that residues 164–286 and 324–475 may behave as distinct structural regions connected by a flexible linker.^43^ Consequently, a two-domain model was constructed in which these regions were treated as independent rigid bodies connected by flexible segments. This representation also provided a direct means of testing the inter-domain rearrangements observed among the BilboMD ensemble conformations. This model was subsequently evaluated using EOM to determine whether inter-domain motions contributed to the observed conformational heterogeneity.

**TABLE V.** Comparison of EOM model statistics for TrhW.

Models	Flexible region	Structure region	#of conformers	χ ^2^	R_g_ ( Å)	D_max_ ( Å)	R_flex_ (%)
Unconstrained	1–475	None	3	1.42	44.6	145.1	78
Single domain	1–163	164–475	5	2.38	43.2	151.4	74
Two-domain	1–163, 287–323	164–286, 324–475	7	1.18	43.7	160.2	80

The two-domain model yielded the best agreement with the experimental SAXS data (χ^2^ = 1.18), substantially improving upon both the unconstrained model (χ^2^ = 1.42) and the single-core constrained model (χ^2^ = 2.38). The selected ensemble consisted of 11 members representing 7 unique conformers and produced an average R_g_ of 43.7 Å, closely matching the experimental value of 44.6 Å. Similarly, the average D_max_ was 160.2 Å, in good agreement with the experimental D_max_ of 160 Å. The selected ensemble exhibited a high degree of conformational heterogeneity (R_flex_ = 80%), indicating that TrhW samples a broad range of conformations in solution. Importantly, the ensemble preserves the overall domain organization predicted by AlphaFold while allowing substantial inter-domain rearrangements that better account for the experimental SAXS data. Together, these results suggest that the experimental scattering data are best described by multiple solution-state conformations rather than a single static structure, with two structured regions connected by flexible segments that permit substantial inter-domain motion while maintaining the overall architecture predicted by AF.

Representative conformers from the selected ensemble illustrate the range of conformations sampled by TrhW in solution [Fig. 7(c)]. Although the overall fold of each structured domain is maintained, the relative orientations of residues 164–286 (TrbC-like domain) and 324–475 (TraW-like domain) vary substantially between conformers, consistent with the inter-domain flexibility suggested by the AF PAE analysis. The selected ensemble occupies a narrower region of conformational space than the initial random pool [Fig. 7(b)] while retaining a high degree of flexibility (R_flex_ = 80%), indicating that TrhW samples multiple solution-state conformations rather than a single rigid structure. Together, these results support a model in which conformational heterogeneity arises from both the flexible N-terminal region and relative motions between the two structured domains connected by a flexible linker.

**FIG. 7.**
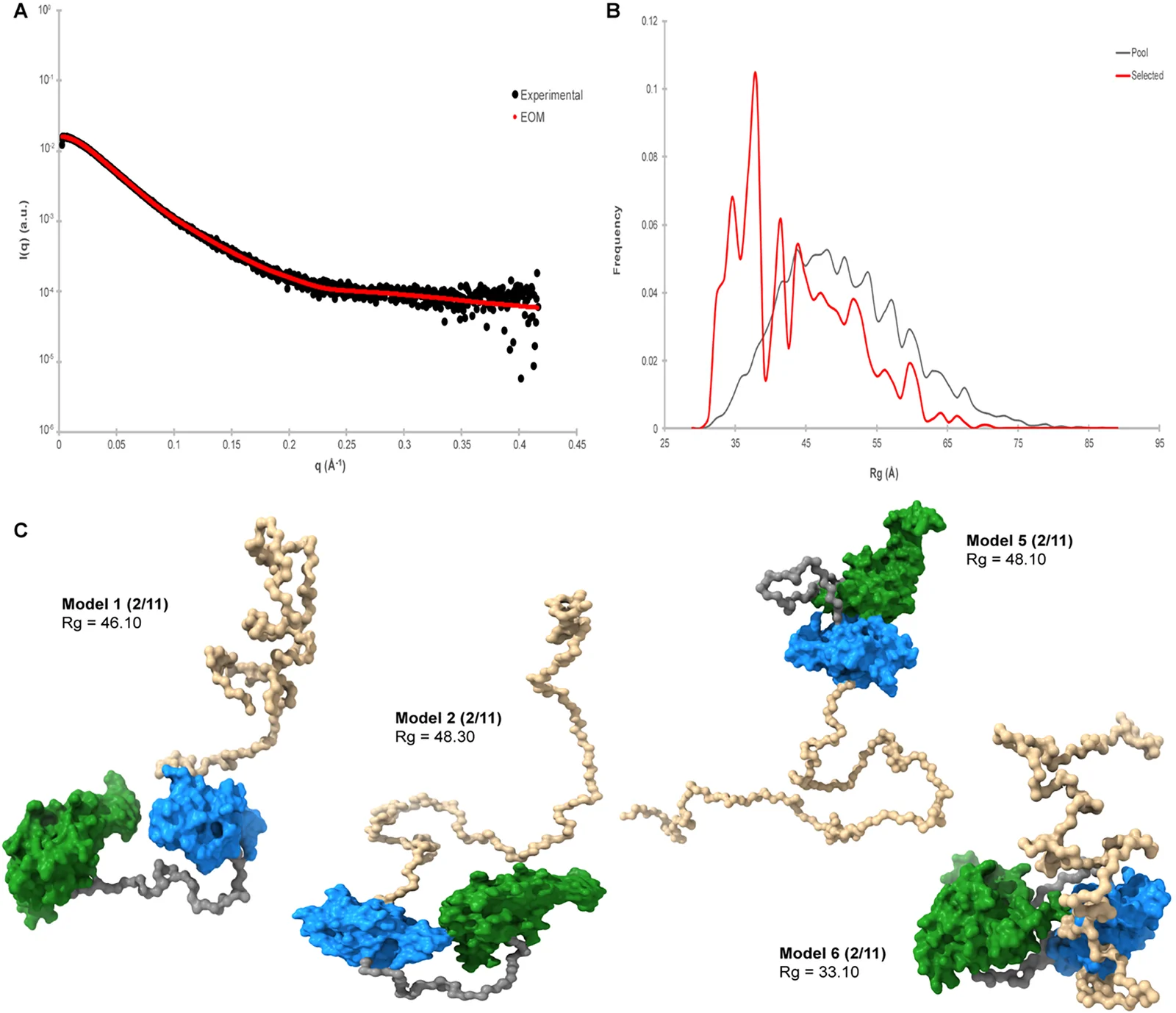
(a) Experimental SAXS scattering profile (black circles) and the best-fit EOM model (red line). The selected ensemble provided excellent agreement with the experimental data ( χ 2 = 1.18). (b) Radius of gyration, R_g_, distributions for the initial random pool generated by RANCH (gray) and the ensemble selected by GAJOE (red). The selected ensemble shifted toward lower R_g_ values relative to the random pool, indicating that the experimental scattering data favor a subset of more compact conformations. (c) Representative conformers from the selected EOM ensemble illustrating the range of solution-state conformations, with each conformer indicated to facilitate comparison with the selected distribution shown in (b). Residues 1–163 are shown in orange, residues 164–286 in blue, 287–323 in gray, and residues 324–475 in green. The structural domains remain conserved while exhibiting variable relative orientations, consistent with inter-domain motion contributing to the conformational heterogeneity observed by SAXS.

The BilboMD and EOM analyses provide complementary descriptions of TrhW conformational heterogeneity. BilboMD showed a substantial improvement in agreement with the experimental scattering data upon transitioning from a single conformation to a multi-state ensemble, with progressively smaller improvements as additional conformers were introduced and little change between the four- and five-state solutions. EOM independently supported this interpretation while providing additional insight into the structural origin of the heterogeneity: treating residues 164–286 and 324–475 as independent structured domains connected by flexible segments produced the best EOM fit and permitted substantial variation in their relative positions and orientations. Although BilboMD and EOM selected ensembles of different sizes, these values should not be interpreted as estimates of the absolute number of discrete conformational states populated by TrhW in solution. Rather, both analyses independently demonstrate that a single static structure is insufficient to describe the experimental SAXS data and support a heterogeneous solution-state ensemble characterized by inter-domain motion while preserving the structures of the individual domains.

Taken together, the complementary biophysical and solution scattering analyses presented here provide a cohesive structural model for TrhW. SEC-MALS established that TrhW exists as a monomer in solution, while CD spectroscopy demonstrated that the protein contains both well-defined secondary structure and a substantial fraction of non-canonical structural elements. SEC-SAXS further revealed that TrhW adopts an elongated architecture with dimensions inconsistent with a compact globular protein, and both BilboMD and EOM analyses demonstrated that the experimental scattering data are best described by multiple conformations rather than a single static structure. Although AlphaFold accurately predicts the overall domain organization of TrhW, the solution-state analyses extend these predictions by demonstrating that the protein undergoes substantial conformational rearrangements in solution while maintaining the overall structures of its individual folded domains. Together, these findings establish TrhW as a dynamic multidomain protein whose conformational heterogeneity is an intrinsic feature of its solution-state behavior and provide a structural framework for future studies investigating its role within the IncHI1 type IV secretion system.

## IV. CONCLUSION

TrhW is a conserved T4SS-associated protein unique to IncHI1 plasmids in that it combines homologous TraW- and TrbC-like regions within a single polypeptide. Using complementary solution-based biophysical and structural approaches, we demonstrate that TrhW exists as a monomeric protein with an elongated architecture and a structured core connected to conformationally dynamic regions. SEC-MALS established its monomeric state, CD spectroscopy revealed a combination of ordered secondary structure and substantial non-canonical structural elements, and SEC-SAXS showed that TrhW adopts an extended solution conformation inconsistent with a compact globular protein. Ensemble modeling using BilboMD and EOM further demonstrated that the experimental scattering data are best explained by multiple solution-state conformations rather than a single rigid structure. Importantly, these analyses indicate that the individual folded domains maintain their overall structures while varying in their relative positions and orientations, supporting a model in which inter-domain motion contributes substantially to the observed conformational heterogeneity. While AlphaFold accurately predicts the overall domain organization of TrhW, the experimental analyses extend these predictions by characterizing its solution-state dynamics and demonstrating that its domain organization is conformationally variable in solution. These findings provide the first experimentally derived structural framework for TrhW and establish a foundation for future studies aimed at defining how its dynamic architecture contributes to the assembly and function of the IncHI1 type IV secretion system.

## Data Availability

The data that support the findings of this study are available from the corresponding author upon reasonable request and are openly available in SASBDB at https://www.sasbdb.org, Ref. 44.
